# Evolutionary dynamics of retrotransposable elements *Rex*1, *Rex*3 and *Rex*6 in neotropical cichlid genomes

**DOI:** 10.1186/1471-2148-13-152

**Published:** 2013-07-16

**Authors:** Carlos Henrique Schneider, Maria Claudia Gross, Maria Leandra Terencio, Edson Junior do Carmo, Cesar Martins, Eliana Feldberg

**Affiliations:** 1 Laboratório de Genética Animal, Instituto Nacional de Pesquisas da Amazônia, Av. André Araújo, 2936, Petrópolis, 69011-970, Manaus, Amazonas, Brazil; 2 Departamento de Biologia, Laboratório de Citogenômica, Instituto de Ciências Biológicas, Universidade Federal do Amazonas, Manaus, AM, Brazil; 3 Departamento de Biologia, Laboratório de Tecnologias de DNA, Instituto de Ciências Biológicas, Universidade Federal do Amazonas, Manaus, AM, Brazil; 4 Departamento de Morfologia, Laboratório Genômica Integrativa, Instituto de Biociências, Universidade Estadual Paulista – UNESP, Botucatu, SP, Brazil

## Abstract

**Background:**

Transposable elements (TEs) have the potential to produce broad changes in the genomes of their hosts, acting as a type of evolutionary toolbox and generating a collection of new regulatory and coding sequences. Several TE classes have been studied in Neotropical cichlids; however, the information gained from these studies is restricted to the physical chromosome mapping, whereas the genetic diversity of the TEs remains unknown. Therefore, the genomic organization of the non-LTR retrotransposons *Rex*1, *Rex*3, and *Rex*6 in five Amazonian cichlid species was evaluated using physical chromosome mapping and DNA sequencing to provide information about the role of TEs in the evolution of cichlid genomes.

**Results:**

Physical mapping revealed abundant TE clusters dispersed throughout the chromosomes. Furthermore, several species showed conspicuous clusters accumulation in the centromeric and terminal portions of the chromosomes. These TE chromosomal sites are associated with both heterochromatic and euchromatic regions. A higher number of *Rex*1 clusters were observed among the derived species. The *Rex*1 and *Rex3* nucleotide sequences were more conserved in the basal species than in the derived species; however, this pattern was not observed in *Rex*6. In addition, it was possible to observe conserved blocks corresponding to the reverse transcriptase fragment of the *Rex*1 and *Rex*3 clones and to the endonuclease of *Rex6*.

**Conclusion:**

Our data showed no congruence between the Bayesian trees generated for Rex1, Rex3 and Rex6 of cichlid species and phylogenetic hypothesis described for the group. Rex1 and Rex3 nucleotide sequences were more conserved in the basal species whereas Rex6 exhibited high substitution rates in both basal and derived species. The distribution of Rex elements in cichlid genomes suggests that such elements are under the action of evolutionary mechanisms that lead to their accumulation in particular chromosome regions, mostly in heterochromatins.

## Background

Transposable elements (TEs) have the potential to produce broad changes in the genomes of their hosts [1,2], acting as a type of evolutionary toolbox and generating a collection of new regulatory and coding sequences [3,4], and have the potential to generate biodiversity and evolutionary transitions through lineage-specific mutations and molecular domestication [5]. In addition, TEs can be transferred between reproductively isolated species through horizontal transfer and can trigger a series of events that actively shapes genomic architecture, as for example through illegitimate recombination between TE copies leading to chromosomal duplications, deletions or inversions, providing raw material for adaptive genomic innovations [6].

Based on their structure and transposition mechanisms, mobile genetic elements are divided into two classes: class I includes transposable elements that have RNA as an intermediate (retrotransposons) that is subsequently copied into cDNA by a reverse transcriptase and integrated into a new genomic site; class II includes the transposons that are directly transposed from DNA to DNA without another intermediate molecule [7-10]. Retrotransposons can also be grouped according to presence or absence of long terminal repeats (LTR). LTRs are necessary for cDNA transcription and integration after reverse transcription; these sequences contain domains for proteinase, integrase, reverse transcriptase, and RNAse. Non-LTR retrotransposons use internal promoters for their transposition and encode a reverse transcriptase and RNAse; these retrotransposons are also known as LINEs (long interspersed elements) or TP (target-primed) retrotransposons. Additionally, SINEs (short interspersed nucleotide elements) fall within the non-LTR category; these retrotransposons do not encode the enzymatic machinery necessary for transposition but probably obtain functionality via LINEs [11].

Various transposable elements are found in genomes and the same type of TE can have very different invasive success in diverse species [11]. TEs have been proposed to be involved in the biodiversity and speciation of Teleost fish. Teleost diversity is also reflected in the diversity of their genome size and structure, which have been considerably affected by retroelements [12,13]. All of the previously described retrotransposon types have been observed in fish genomes [14-17], and among the non-LTR retrotransposons, *Rex*1, *Rex*3, and *Rex*6 were active during the evolution of teleost fish [16,18,19]. The *Rex*1 TE is related to the *CR*1 clade of LINEs and encodes a reverse transcriptase, which is frequently removed by incomplete reverse transcription [18]. *Rex*3 is related to the RTE family and essential features of the element in fish are (i) coding regions for an endonuclease and a reverse transcriptase, (ii) 5’ truncations of most of the copies, (iii) a 3’ tail consisting of tandem repeats of the sequence GATG, and (iv) short target site sequence duplications of variable length [16]. *Rex*6 encodes a reverse transcriptase and a putative restriction enzyme-like endonuclease and is a member of the *R*4 family of non-LTR retrotransposons [16].

Fish belonging to the family Cichlidae (Perciformes) are considered an excellent evolutionary model due to their adaptive radiation and their ecological and behavioral diversity [20,21]. Among the cichlids, only the subfamily Cichlinae is found in the Neotropical region. Analysis combining the morphological and nucleotide sequence characteristics allowed the subfamily Cichlinae to be recovered as monophyletic and partitioned into seven tribes: Cichlini (basal tribe), Retroculini, Astronotini, Chaetobranchini, Geophagini, Cichlasomatini, and Heroini (derived tribe). Chaetobranchini and Geophagini was resolved as the sister group of Heroini and Cichlasomatini. The mono-generic Astronotini was recovered as the sister group of these four tribes. Finally, a clade composed of Cichlini and Retroculini was resolved as the sister group to all other cichlines [22]. To date, 135 species of cichlids have been cytogenetically analyzed, the diploid number of chromosome is predominantly 2n = 48 (more than 60% of the studied species), although variations ranging from 2n = 38 to 2n = 60 have been described. For African species, the modal diploid number of chromosome is 2n = 44. In the other hand, for Neotropical cichlids, the most common chromosome number is 2n = 48, which is considered to be the ancestral characteristic for all cichlids [23,24].

In the Amazon region some species of cichlids are important in recreational and subsistence fishing, aquaculture, and aquarium-hobby, as *Cichla monoculus* (Cichlini), *Astronotus ocellatus* (Astronotini), *Geophagus proximus* (Geophagini), *Pterophyllum scalare* and *Symphysodon discus* (Heroini). *Cichla* species have karyotypes with 2n = 48 subtelo/acrocentric (st/a) chromosomes, few heterochromatin and nucleolus organizer regions located on one chromosome pair [25]. This pattern is described as basal to the Neotropical cichlids [23,26]. Although *A. ocellatus*, *G. proximus* and *P. leopoldi* also have 2n = 48 chromosomes, they differ in karyotype formula, with meta/submetacentric (m/sm) chromosomes due to chromosomal inversions, and accumulation of heterochromatin in the pericentromeric regions [27]. The highest diploid number described for Cichlinae is found in species of the genus *Symphysodon*, which has 2n = 60 chromosomes, as well as large heterochromatic blocks. These heterochromatic regions are rich in the *Rex*3 TE that seems to have been active in the karyotype evolution of *Symphysodon*, being probable involved in translocation events [28].

Several TE classes were cytogenetically mapped on the Neotropical cichlid genome: Tc*1* transposons in *Cichla kelberi,* which showed conspicuous blocks in the centromeric regions and small clusters scattered throughout the chromosomes [29]; *RCk*, which exhibits clusters scattered throughout the *C. kelberi* chromosome; Ao*Rex*3 and AoLINE, which are clustered in the centromeric heterochromatin of all of the chromosomes of the complement in *Astronotus ocellatus*[29]; and *Rex* elements were mapped in few species [28-31]. Furthermore, these findings are restricted to physical chromosome mapping, and the genetic diversity of these retroelements remains unknown.

*Rex*1, *Rex*3 and *Rex*6 are widely distributed among fish genomes, thus enabling comparative analysis among species. Therefore, comparative analyses encompassing basal and derived species are of particular interest for understanding the role of retroelement dynamics in the genomic evolution of new world cichlids. This study aimed to evaluate the genomic organization of three non-LTR retrotransposons, *Rex*1, *Rex*3, and *Rex*6, in five Amazonian cichlids of different tribes, comprising basal and derived species, using the combination of physical chromosome mapping and DNA sequencing in the way to provide information about the role of TEs in the evolution of cichlid genomes.

## Results

### Physical chromosome mapping of TEs

**Figure 1:**
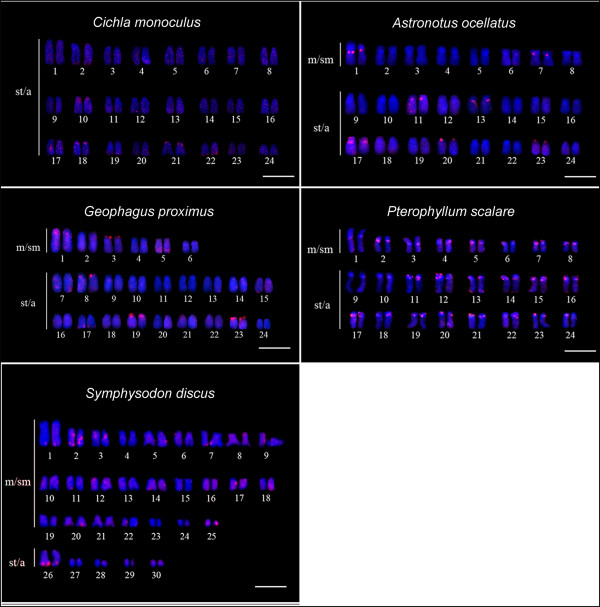
**Cytogenetic mapping of *Rex*1 retrotransposon (red signals).** Chromosomes were counterstained with DAPI. m/sm = meta/submetacentric chromosomes; st/a = subtelo/acrocentric chromosomes. Bar = 10 μm.

For a better comparison of the physical chromosome location of Rex retroelements in relation to the pattern of constitutive heterochromatin, chromosome distribution of telomeric sequences, and ribosomal sites described for the five species, we followed karyotypic organization recently proposed for these species [27]. The chromosomes were separated into classes (meta/submetacentric – sm/sm, and subtelo/acrocentric – st/a), matched on the basis of hybridization patterns and organized in descending order of size. The *Rex*1 retroelement was scattered throughout the chromosomes of *Cichla monoculus, Astronotus ocellatus*, *Geophagus proximus*, *Pterophyllum scalare*, and *Symphysodon discus*. Furthermore, *Astronotus ocellatus* exhibited pairs 11, 17, and 23 with extensive distribution of this retroelement; similar results were observed for pairs 1, 5, 7, 8, 14, 15, 19, 20, 21 and 23 of *Geophagus proximus* and for most of the chromosome pairs of *Symphysodon discus.* However, all the species exhibited more conspicuous clusters in the terminal and/or centromeric regions with more markings in *Pterophyllum scalare* (Figure 1).

**Figure 2:**
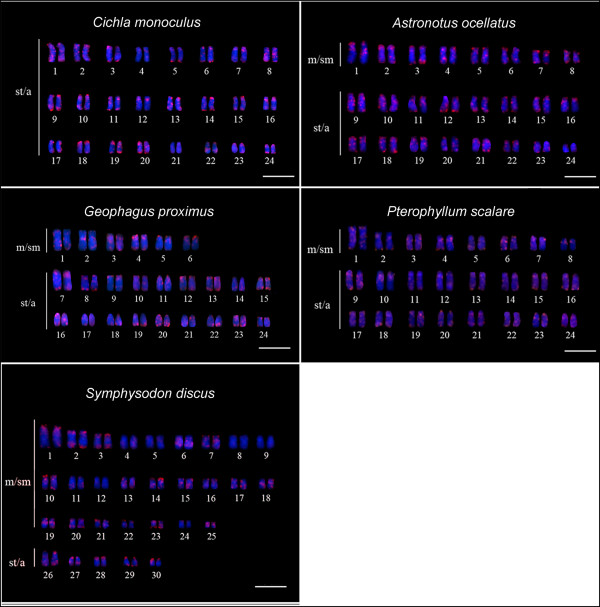
**Cytogenetic mapping of *Rex*3 retrotransposon (red signals).** Chromosomes were counterstained with DAPI. m/sm = meta/submetacentric chromosomes; st/a = subtelo/acrocentric chromosomes. Bar = 10 μm.

**Figure 3:**
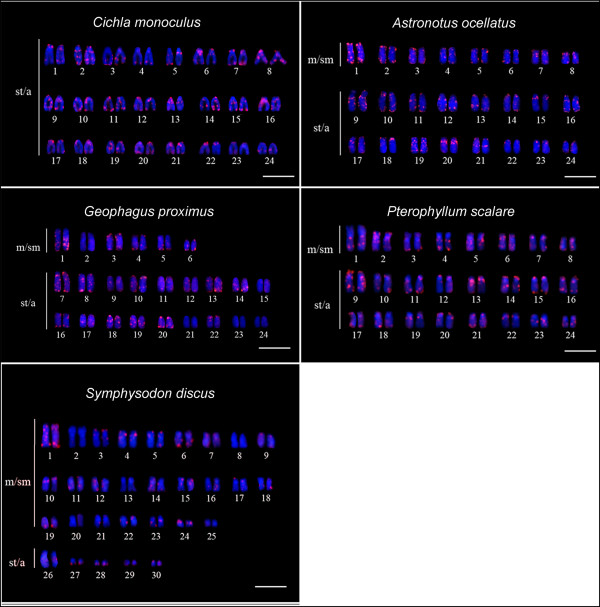
**Cytogenetic mapping of *Rex*6 retrotransposon (red signals).** Chromosomes were counterstained with DAPI. m/sm = meta/submetacentric chromosomes; st/a = subtelo/acrocentric chromosomes. Bar = 10 μm.

Multiple and intense sites of hybridization were obtained for the *Rex*3 and *Rex*6 retroelements in the five species assessed. These TEs appear to have an almost pan-genomic distribution with larger clusters in terminal regions of most chromosomes from the five species, as well as in the centromeric, pericentromeric and interstitial regions. In the other hand, few unlabeled chromosomes were visualized (Figures 2 and 3).

**Figure 4:**
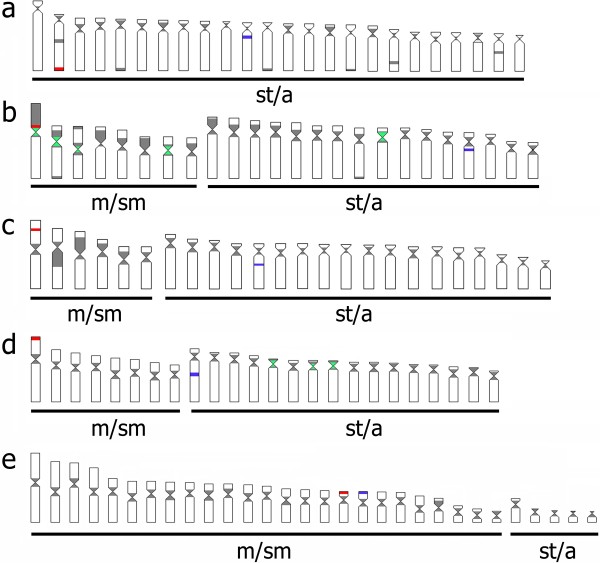
**Representative idiograms of Neotropical cichlid species.** (**a**) *C. monoculus*, (**b**) *A. ocellatus*, (**c**) *G. proximus*, (**d**) *P. scalare*, (**e**) *S. discus*; in gray heterochromatic regions; in red 18S rDNA; in blue 5S rDNA; green, interstitial telomeric sites (ITS).

Based in the comparative analysis of the cytogenetic mapping results obtained in the present work with heterochromatin/euchromatin distribution previously published for the same species [25,27-29] it is clear that most *Rex* clusters are indeed located in heterochromatic chromosomal areas. However, small clusters of *Rex*1, *Rex*3 and *Rex*6, seem also to be located in euchromatic areas (Figure 4).

### Molecular diversity of TEs

**Figure 5:**
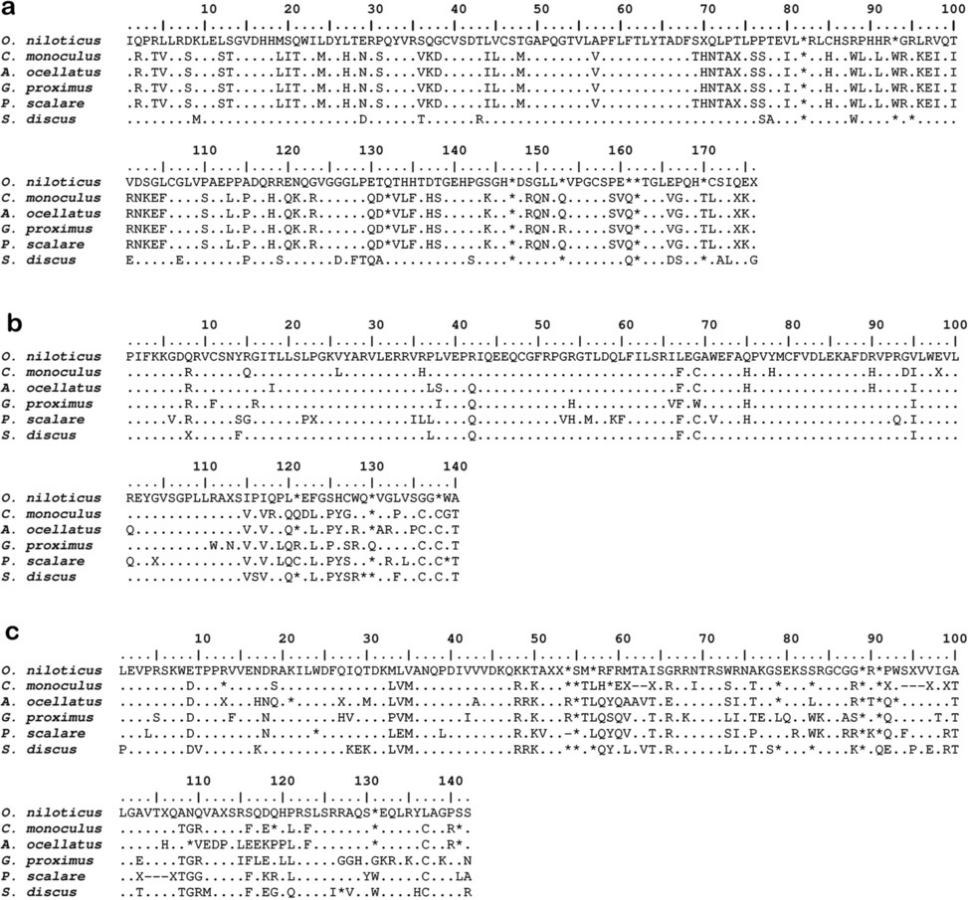
**Alignment of aminoacid sequences of TEs among cichlids**: **a**) *Rex*1 reverse transcriptase domain of analyzed species compared with *O. niloticus* (accession number AJ288473); **b**) *Rex*3 reverse transcriptase domain compared with *O. niloticus* (accession number AJ400370); **c**) *Rex*6 endonuclease domain of analyzed species compared with *O. niloticus* (accession number AJ293545). Dots indicate similarity in sequence and asterisks indicate stop codons.

**Figure 6:**
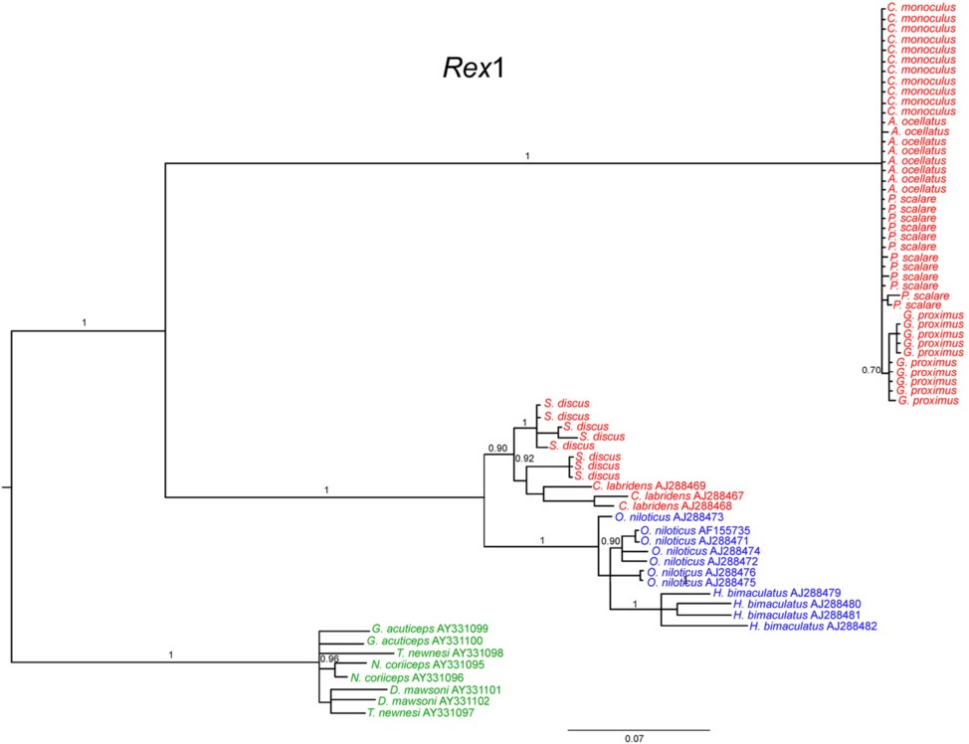
**Bayesian tree for reverse transcriptase nucleotide sequences revealing two monophyletic lineages of *Rex*1 retroelement.** Numbers above the branches represent Bayesian posterior probability values. In red Neotropical cichlid species; in blue African cichlid species; in green Perciformes marine species.

A total of 49 *Rex*1 clones were sequenced: 11 of *C. monoculus*, 8 of *A. ocellatus*, 10 of *G. proximus*, 12 of *P. scalare*, and 8 of *S. discus*. Sequences of 573- to 575-bp (base pairs) long were obtained. Nucleotide substitutions were observed more frequently in the derived species (*P. scalare* and *S. discus*) and less so in the basal species (*C. monoculus* and *A. ocellatus*). No substitutions were observed in *C. monoculus*, whereas the following nucleotide substitutions were observed in the other species: 1 transition in *A. ocellatus*; 2 transitions in *G. proximus*; 2 transitions and 3 transversions in *P. scalare*; and 18 transitions, 7 transversions, and 4 indels (insertions/deletions) in *S. discus*. Compared with the *Rex*1 sequence of other cichlids available in Genbank, a 91% genetic identity with *Hemichromis bimaculatus* [GenBank: AJ288479] and a 94% genetic identity with *Oreochromis niloticus* [GenBank: AJ288473] were observed. It was also possible to identify conserved blocks corresponding to the reverse transcriptase coding region, with corresponding similarity between *S. discus* and *O. niloticus*, as well as similarity among the others (Figure 5a). The phylogenetic tree of *Rex*1 element clones indicated separation between *Symphysodon discus* and the remaining species, as indicated by two branches (Figure 6). These tree clustering do not reflect the phylogenetic relationship of the species assessed. However, all cichlid *Rex*1 sequences form a monophyletic group (containing two branches) divergent from the marine Perciformes species. The branch 1: grouped *S. discus* and *Cichlasoma labridens*, (both Neotropical cichlids), and form a sister group with *Oreochromis niloticus* and *Hemichromis bimaculatus* (African cichlids). The branch 2: encompasses sequences of *C. monoculus*, *A. ocellatus*, *P. scalare* and *G. proximus*. The average genetic distance between species varied from 0.33 to 0.68% among *C. monoculus*, *A. ocellatus*, *P. scalare* and *G. proximus*. This value is lower compared to the data detected by comparing these species with *S. discus*, which ranged from 36.37 to 37.06%. The latter are similar to the average genetic distance between branch 2 (*C. monoculus*, *A. ocellatus*, *P. scalare* and *G. proximus*) and African cichlids, which ranged from 34.95 to 38.11% (Additional file 1).

**Figure 7:**
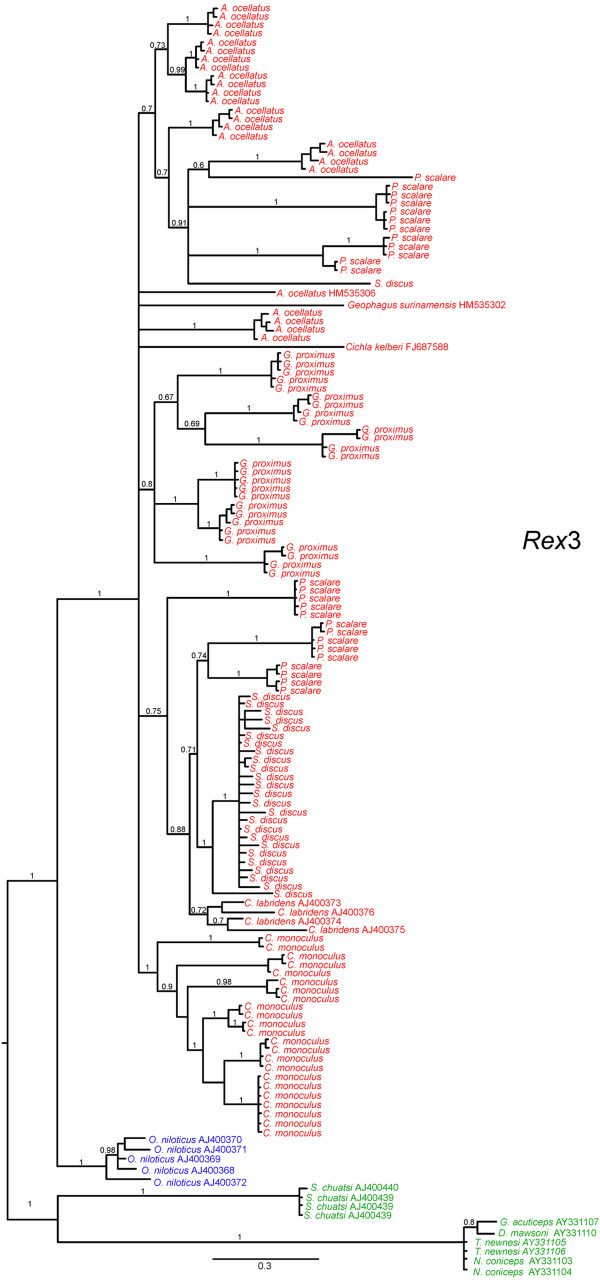
**Bayesian tree for reverse transcriptase nucleotide sequences of *Rex*3 retroelement.** Numbers above the branches represent Bayesian posterior probability values. In red Neotropical cichlid species; in blue African cichlid species; in green Perciformes marine species.

Sequences of 418-475 bp in length were obtained from the 126 *Rex*3 clones assessed. There were a total of 23 clones of *C. monoculus*, 24 of *A. ocellatus*, 27 of *G. proximus* and 26 clones of *P. scalare* and *S. discus*. The *Rex*3 retroelement was highly variable in all of the species assessed with the following multiple nucleotide substitutions: 114 in *C. monoculus* (79 transitions, 25 transversions, and 10 indels), 159 in *A. ocellatus* (83 transitions, 19 transversions, and 57 indels), 215 in *G. proximus* (89 transitions, 34 transversions, and 92 indels), 242 in *P. scalare* (130 transitions, 58 transversions, and 54 indels), and 245 in *S. discus* (133 transitions, 58 transversions, and 54 indels). When compared with *Rex*3 sequences from other cichlids available in Genbank, an 87% genetic identity with *Oreochromis niloticus* [GenBank: AJ400370], a 91% genetic identity with *Cichla kelberi* [GenBank: FJ687588], and a 92% genetic identity with *Geophagus surinamensis* [GenBank: HM535302] were observed. Even with wide variations in the cloned sequences, it was possible to identify conserved blocks corresponding to the *Rex*3 reverse transcriptase coding sequence (Figure 5b). *Rex*3 sequences of marine Perciformes clustered separated from all cichlids species, and *O. niloticus* (African cichlid) appeared as sister group of the South American cichlids. With exception of *C. monoculus*, the other species formed paraphyletic branches and were not grouped into exclusive clades (Figure 7). Regarding the genetic distance between species, the values ranged from 6.96 to 16.35% in Neotropical cichlids, values which are close to those found when compared with African cichlids (Additional file 2).

**Figure 8:**
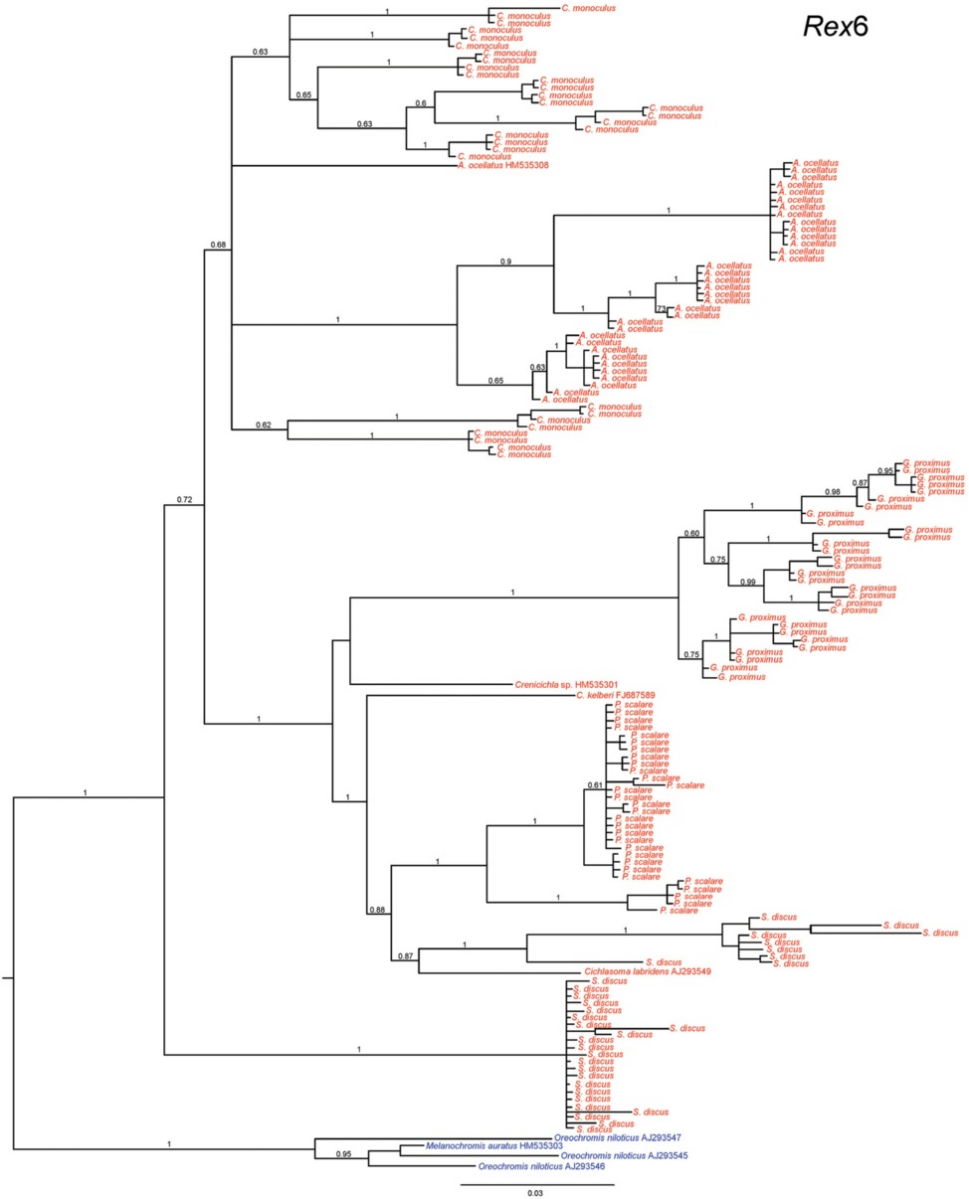
**Bayesian tree for endonuclease nucleotide sequences of *Rex*6 retroelement.** Numbers above the branches represent Bayesian posterior probability values. In red Neotropical cichlid species; in blue African cichlid species.

Fragments between 459 and 505 bp in length were generated for the 156 *Rex*6 retroelement cloned sequences. A total of 30 *C. monoculus* clones, 34 *A. ocellatus* clones, 30 *G. proximus* clones, 30 *P. scalare* clones, and 32 *S. discus* clones were sequenced. In addition, *C. monoculus* and *S. discus* showed high nucleotide substitution rates: 214 (81 transitions, 63 transversions, and 70 indels) and (103 transitions, 88 transversions, and 23 indels), respectively. Nucleotide substitutions were also observed in the other species: 78 (48 transitions and 19 transversions and 11 indels) in *G. proximus,* 67 (41 transitions, 17 transversions, and 9 indels) in *A. ocellatus* and 54 (36 transitions, 7 transversions, and 11 indel) in *P. scalare*. When compared with the other *Rex*6 cichlid sequences available in Genbank, the following sequence identities were observed: an 81% identity with *Oreochromis niloticus* [GenBank: AJ293545], an 83% identity with *Melanochromis auratus* [GenBank: HM535303], and an 88% identity with *Crenicichla* sp. [GenBank: HM535301]. The sequences exhibited a region of homology with the *Rex*6 endonuclease domain (Figure 5c). The pairwise genetic distance between species ranged from 6.52% (between the African species *Melanochromis* sp. and *Oreochromis* sp) to 24.63% (observed between Neotropical species *A. ocellatus* and *S. discus*) (Additional file 3). The clones of each species were grouped into distinct branches on the phylogenetic tree for the *Rex*6 element in which *A. ocellatus* appears grouped of all *C. monoculus* clones. African cichlids form a sister group to all South American species. All *C. monoculus* and *A. ocellatus* clones formed a sister group to the remaining derived species. *Geophagus proximus* formed a sister group of *Crenicichla* sp., which is a basal clade to the one that includes *P. scalare, S. discus* and *Cichlasoma labridens*. In addition, two lineages of *Rex*6 are evident in *S. discus*. One lineage related to sequences of *C. labridens* and another forming the basal group to the all analyzed species (Figure 8).

## Discussion

### Chromosomal distribution of TEs clusters

Recent studies have indicated that cichlids have a dynamic karyotype, with variations being observed in the chromosome number, karyotype structure, and in relation to the distribution of repetitive DNA, with differences in the number and location of rDNA sites, presence of interstitial telomeric sites in some taxa and pattern of TEs distribution among species, which vary between dispersed and compartmentalized [24,27,28,31]. Among the repetitive elements, TEs stand out due to their ability to generate evolutionary change through various processes, including gene inactivation, the combining of exons, and gene conversion [for review, see [32]. In addition, ectopic homologous recombination between nonallelic copies of TEs can lead to the formation of deletions, duplications, inversions and translocations, and the process of transposition itself can induce various types of rearrangements at the target site [13]. It has long been known that cryptic and non-autonomous TEs accumulate in heterochromatic regions of the genome, including in fish [27-29,31,33]. However, in certain species, these transposable elements are found in euchromatic regions [28,30,34]. Unlabeled chromosomes visualized for *Rex*3 and *Rex*6 suggests that the TEs could have been lost due to molecular processes that erode repetitive DNA sequences, or to the presence of few TE residues could have escaped detection by FISH. All specimens here investigated had their heterochromatic pattern previously described [27] and presented accumulation of *Rex*1, *Rex*3, and *Rex*6 clusters mostly in heterochromatic regions, but small clusters were also detected in euchromatic areas. This result indicates at least two hypotheses: i) the chromosomal distribution of these TEs can be driven by particular evolutionary mechanisms and may evolve differentially from other repetitive sequences commonly present in the heterochromatin; ii) these TEs could have acquired structural/regulatory functions and their movement to euchromatic regions could bring advantage to the host genome or have been maintained according to neutral mechanisms.

The movement and accumulation of TEs has a great influence of host genomes, and it is becoming clear that TEs form part of the regulatory toolkit of the genome and play important roles in controlling gene expression [35,36], although the relationship between TEs and regulatory function is not always straightforward and easily visualized. Nevertheless, correlation between karyotypic rearrangements and retrotransposon activity was observed in the marine Perciformes, for which compartmentalized distribution with accumulation in the pericentromeric regions is observed among the derived species, such as *Notothenia coriiceps*[37].

Chromosomal rearrangements are evident in Cichlinae evolution, and interstitial telomeric sites (ITS) are recognized in *Astronotus ocellatus*[27]. These ITS are probably associated with various classes of repetitive DNA, and these TEs may have possibly acted in the chromosomal rearrangements of *A. ocellatus* because the retroelements *Rex*1, 3, and 6 appear in the same chromosomal regions where the ITSs were observed [27]. The accumulation of TEs in particular genomic regions of cichlids has been associated to possible events of chromosome rearrangements [28-31,38].

TEs can insert into virtually any portion of the host genome; however, certain preferred insertion regions have already been reported, such as in ribosomal genes [39,40]. An association between *Rex*1, *Rex*3, and *Rex*6 and the 18S and 5S ribosomal sites was observed in all of the species assessed in this study. This association between TE and rDNA in cichlids may have had a role in the generation of multiple 18S rDNA sites found in *P. scalare*[27] and the 18S rDNA site variability observed in *Symphysodon aequifasciatus*[41]. Analysis of the 18S rRNA gene distribution in the *Oreochromis niloticus* genome reveals that 18S copies are always surrounded by TEs, suggesting that such elements could be acting in the dispersion of 18S copies generating higher number of clusters and sites variability [42].

### Diversity and evolution of *Rex*1, *Rex*3 and *Rex*6 TEs

Although little is known about the genomic evolution and the effects of retroelements in fish, these elements appear to be responsible for the karyotype dynamism that has been recently revealed in cichlids [23,24,27,28,30]. This dynamism is also exemplified by an examination of Neotropical cichlid TE sequences. *Rex*1 exhibited two evolutionary lineages, even being the most conserved TE with few nucleotide substitutions. High values of genetic distance were found for *Rex*1 among Neotropical species (*C. monoculus, A. ocelatus, G. proximus* and *P. scalare*) and (*C. labridens* and *S. discus*). But the genetic distance was lower when compared *S. discus* (Neotropical species) and *O. niloticus* (African species).

The presence of various *Rex*1 lineages has been observed previously in fish genomes with four reported lineages for this retroelement [18]. The clones assessed in this study correspond to lineage 4, which is commonly observed in the genome of fish from superorder Acanthopterygii [18].

The emergence of lineages may be the result of the frequent loss or rapid divergence of these retroelements [43]. In addition, the high level of similarity among the *Rex* sequences of some teleost fish species suggests relatively recent activity of this retroelement [19]. However, the *Rex* sequences of cichlids showed a high degree of variability and *Rex*3 showed a paraphiletic Bayesian tree that does not reflect the phylogeny proposed for the group. In this case, probably several *Rex*3 strains invaded the genome of these species and have been retained, with gain and loss of sequences within each species. Furthermore, the presence of stop codons in the TE aminoacid sequences suggests that these sequences correspond to inactive elements. If considered the group evolutionary history, perhaps *Rex* TEs have acted shaping karyotypes, but are no longer active. This fact can be reinforced because it is possible to observe conserved blocks corresponding to the reverse transcriptase domain of *Rex*1 and *Rex*3 and to the endonuclease for *Rex*6.

*Rex*1 and *Rex*3 nucleotide sequences were more conserved in the basal species (*C. monoculus*) than in the derived species (*S. discus*). This result is possibly associated with the selective forces that tend to stabilize the genome, which frequently eliminates TE families from the genome of some species [11], as the repression mechanisms in each host may be different [44]. Furthermore, the genomic evolution of *Symphysodon* is complex and involves hybridization events among the species [45], which may have caused instability in the TEs and led to transposition events that resulted in multiple chromosomal rearrangements [28]. In contrast, *Rex*6 exhibited high substitution rates in *C. monoculus* (basal clade) that were similar to those observed in *S. discus* (derived). *Rex*6 presents a remarkable characteristic with two clearly defined subfamilies in *S. discus*. The accumulation of mutations may be associated with TE senescence, which is usually characterized by an accumulation of mutations followed by the probable stochastic loss of the element [46].

Studies have demonstrated that TE sequences evolve independently in the genomes of their hosts [47,48], and this evolutionary pattern can be a consequence of horizontal transfer, which causes incongruence between the host and TE phylogenies [49]. In this view, the phylogenetic trees of *Rex*1, *Rex*3 and *Rex*6 do not reflect the species phylogenetic tree [22], and it is likely that horizontal transfer events occurred in these retroelements during the evolutionary history of the species that were assessed. Although previous studies have suggested that *Rex* elements could have suffered the action of horizontal transfer [30,37], ancestral polymorphism and different TE evolutionary rates among the hosts, including loss and gain of TE copies, cannot be disregarded. Although advances in undertanding the evolutionary dynamics of TEs in cichlids have been achieved, the increase in sampling species and sequences is still necessary to have a better view of the organization and function of TEs in the fish genomes.

## Conclusions

The distribution of *Rex* elements in cichlid genomes suggest that such elements are under the constraining of evolutionary mechanisms that lead to their accumulation in particular chromosome regions (mostly in heterochromatins). There was no congruence between the trees generated in this study based on TEs and proposed phylogenetic hypothesis for the group, suggesting that horizontal transfer, emergence or elimination of specific TE lineages, could have had important effects in the evolutionary history of *Rex* elements in the cichlid genomes.

## Methods

Specimens belonging to four Cichlidae tribes of the subfamily Cichlinae from the central Amazon region were analyzed. The tribes included Cichlini: *Cichla monoculus* (3 males and 3 females), Astronotini: *Astronotus ocellatus* (3 males and 5 females), Geophagini: *Geophagus proximus* (2 males and 3 females), and Heroini: *Pterophyllum scalare* (3 males and 3 females) and *Symphysodon discus* (2 males and 2 females). The tribes sampled include basal and derived clades [22]. The specimens were caught in the wild with sampling permission (ICMBio SISBIO 10609-1/2007). Basic kayotypic analyses with the same s species were previously conducted to describe the diploid number, karyotype formula, pattern of constitutive heterochromatin distribution, number and location of ribosomal sites and sequences telomeric [27].

### Chromosomes preparation

Mitotic chromosomes were obtained from kidney cells using an air drying protocol [50], that consists in injecting intraperitoneally colchicine 0.0125% in the proportion of 1 mL per 100 g of animal weight. After 40 minutes the specimens were killed by immersion in ice water and the anterior portion of the kidney was removed, and transferred to a plate with 8 mL of hypotonic solution of 0.075 M KCl. The tissue was disaggregated with a glass syringe and the supernatant (cell suspension) was transferred to a centrifuge tube and incubated at 37°C for 30 minutes. The suspension was pre-fixed with 6 drops of Carnoy fixative (methanol: acetic acid 3:1) and resuspended. After 5 minutes, itwas added 8 mL of Carnoy and centrifuged for 10 minutes at 900 rpm. The supernatant was discarded and 6 mL of Carnoy was added. The material was resuspended and again centrifuged for 10 minutes at 900 rpm, and this wash was repeated twice. After the last centrifugation and removal of supernatant, 1.5 mL of fixative was added and the material carefully resuspended. The cell suspension was then stored in a 1.5 mL Eppendorf tube and stored in a freezer at -20°C.

### DNA extraction and polymerase chain reaction (PCR) amplification

Total DNA was extracted from muscle tissue using phenol-chloroform [51] and quantified in a NanoVue Plus spectrophotometer (GE Healthcare). Polymerase chain reaction (PCR) amplifications of the retroelements were conducted using the primers RTX1-F1 (5’-TTC TCC AGT GCC TTC AAC ACC-3’) and RTX1-R1 (5’-TCC CTC AGC AGA AAG AGT CTG CTC-3’) to amplify the *Rex*1 segments corresponding to the coding domains of the reverse transcriptase gene [18]; the primers RTX3-F3 (5’-CGG TGA YAA AGG GCA GCC CTG-3’) and RTX3-R3 (5’-TGG CAG ACN GGG GTG GTG GT-3’) were used to amplify the coding domains of the reverse transcriptase gene of the *Rex*3 retrotransposon [16]; the primers *Rex*6-Medf1 (5’-TAA AGC ATA CAT GGA GCG CCA C-3’) and *Rex*6-Medr2 (5’-GGT CCT CTA CCA GAG GCC TGG G-3’) were designed to amplify the C-terminal part of the endonuclease domain of the *Rex*6 retrotransposon [19]. All primers were first described for *Xiphophorus maculatus.* The PCR reactions were performed in a final volume of 25 μL containing genomic DNA (200 ng), 10x buffer with 1.5 mM magnesium, Taq DNA polymerase (5 U/μL), dNTPs (1 mM), primers (5 mM), and Milli-Q water. The cycling conditions for the *Rex*1, *Rex3*, and *Rex6* reactions included the following steps: 95°C for 5 min; 35 cycles of 95°C for 1 min, 55°C for 40 s, and 72°C for 2 min; and a final extension at 72°C for 5 min. The PCR products were analyzed using electrophoresis in 1% agarose gels, quantified in a spectrophotometer NanoVue Plus (GE Healthcare) and used for cloning and as probes to perform FISH.

### Cloning, sequencing and sequence analysis

The PCR products of the retrotransposon elements *Rex*1, *Rex3,* and *Rex6* were inserted in the plasmid pGEM-T Easy (Promega). Ligation products were transformed into DH5α *Escherichia coli* competent cells. Clones carring the insert of interest were sequenced on an ABI 3130 XL DNA sequencer (Perkin-Elmer), and the resulting sequences were submitted to the NCBI database under the following accession numbers: *Rex*1, GenBank: JX576302-JX576350; *Rex*3, GenBank: JX576351-JX576400; KF131681-KF131756; and *Rex*6, GenBank: JX576401-JX576459; KF131757-KF131853. Each clone was used as a query in BLASTn searches against the NCBI nucleotide collection (http://www.ncbi.nlm.nih.gov) and the searches against the Repbase [52] at the Genetic Information Research Institute (Giri) (http://www.girinst.org/repbase/) using CENSOR software [53]. Nucleotide sequences were aligned using the ClustalW program package [54] implemented in the BioEdit 7.0 program package [55]. A Bayesian phylogenetic analysis was conducted using MrBayes 3.2 [56]. For this analysis, Markov Chain Monte-Carlo sampling was conducted every 20000th generation until the s.d. of split frequencies was <0.01. A burn-in period equal to 25% of the total generations was required to summarize the parameter values and trees. Parameter values were assessed based on 95% credibility levels to ensure the analysis had run for a sufficient number of generations. For Bayesian analysis, sequences of the *Rex*1, *Rex*3, and *Rex*6 retroelements of all Perciformes (corresponding to the TE segment isolated for cichlids by PCR), available in GenBank, were included. To estimate divergence between species, a genetic distance matrix was constructed using the MEGA5 program and Kimura-2 parameter model [57]. Aminoacid sequences were deduced from nucleotide sequences using BioEdit 7.0 program package [49].

### Fluorescence in situ hybridization (FISH)

The retroelements (*Rex*1, 3, and 6) were labeled with digoxigenin-11-dUTP (Dig- Nick Translation mix; Roche) by nick translation according to the manufacturer’s instructions, and anti-digoxigenin rhodamine (Roche) antibody was used to detect the probe signal. Homologs and heterologs fluorescence in situ hybridizations were carried with 77% stringency (2.5 ng/μL of DNA, 50% deionized formamide, 10% dextran sulfate, and 2x SSC at 37°C for 18 h) [58]. The chromosomes were counterstained with DAPI (2 mg/ml) in Vectashield mounting medium (Vector). Four slides and a minimum of 30 metaphases were analyzed per species.

### Microscopy/image processing

Chromosomes were analyzed in objective of 100x using an Olympus BX51 epifluorescence microscope, and the images were captured with a digital camera (Olympus DP71) using the Image-Pro MC 6.3 software. Mitotic metaphases were processed in Adobe Photoshop CS3 program, and the chromosomes were measured using Image J. Karyotypes were arranged in order of decreasing chromosome size [59].

## Competing interests

The authors declare that they have no competing interests.

## Authors’ contributions

CHS, MCG and MLT collected the samples, collaborated on all genetic procedures, undertook the bibliographic review, and coordinated the writing of this paper. EJC participated in developing the laboratory techniques performed cloning of TEs. CM and EF coordinated the study, participated in its design and revised the manuscript. All authors read and approved the final manuscript.

## Supplementary Material

Additional file 1 **Kimura-corrected average pairwise distances (intersection between line and column) between aligned sequence of *Rex*1 partial reverse transcriptase sequences from Cichlids and Perciformes marine species.** Above diagonal, average genetic distance within species. Click here for file

Additional file 2 **Kimura-corrected average pairwise distances (intersection between line and column) between aligned sequence of *Rex*3 partial reverse transcriptase sequences from Cichlids and Perciformes marine species.** Above diagonal, average genetic distance within species. Click here for file

Additional file 3 **Kimura-corrected average pairwise distances (intersection between line and column) between aligned sequence of Rex6 partial endonuclease from Neotropical and African species.** Above diagonal, average genetic distance within species. Click here for file
