# Supplementary material for: Evolutionary dynamics of retrotransposable elements Rex1, Rex3 and Rex6 in neotropical cichlid genomes
Source: BMC Evol Biol. 2013 Jul 16;13:152. doi: 10.1186/1471-2148-13-152 (PMC3728117; doi:10.1186/1471-2148-13-152)
Supplement: Additional file 1 — Kimura-corrected average pairwise distances (intersection between line and column) between aligned sequence of Rex1 partial reverse transcriptase sequences from Cichlids and Perciformes marine species. Above diagonal, average genetic distance within species. [file 1471-2148-13-152-S1.pdf]

Additional file 1. Kimura-corrected average pairwise distances (intersection between line and column) between aligned sequence of *Rex*1 partial reverse transcriptase sequences from Cichlids and Perciformes marine species. Above diagonal, average genetic distance within species.

	Cichlid species								Perciformes marine species			
	Neotropical					African						
	C. monoculus	*A. ocellatus*	*G. proximus*	*P. scalare*	*S. discus*	*C. labridens*	*O. niloticus*	*H. bimaculatus*	*Dissostichus* sp.	*Notothenia* sp.	*Trematomus* sp.	*Gymnodraco* sp.
	0	0.10%	0.20%	0.20%	2.10%	3.60%	1.60%	4.50%	0	1.10%	4.50%	3.40
C. monoculus												
A. ocellatus	0.47%											
G. proximus	0.35%	0.38%										
P. scalare	0.33%	0.36%	0.68%									
S. discus	37.06%	36.37%	36.87%	36.54%								
C. labridens	37.65%	36.94%	37.46%	37.14%	4.45%							
O. niloticus	35.64%	34.95%	35.47%	35.21%	7.61%	9.00%						
H. bimaculatus	38.11%	37.41%	37.92%	37.62%	9.70%	11.16%	4.78%					
Dissostichus sp.	35.86%	35.86%	36.40%	35.42%	24.53%	25.13%	27.37%	29.20%				
Notothenia sp.	37.39%	37.39%	37.94%	36.93%	24.09%	24.84%	26.45%	28.18%	3.84%			
Trematomus sp.	37.95%	37.95%	38.50%	37.49%	24.31%	25.13%	26.62%	28.64%	3.84%	4.07%		
Gymnodraco sp.	37.85%	37.85%	38.40%	37.39%	24.63%	25.30%	27.24%	29.04%	4.31%	2.92%	3.95%	
