# Supplementary material for: Evolutionary dynamics of retrotransposable elements Rex1, Rex3 and Rex6 in neotropical cichlid genomes
Source: BMC Evol Biol. 2013 Jul 16;13:152. doi: 10.1186/1471-2148-13-152 (PMC3728117; doi:10.1186/1471-2148-13-152)
Supplement: Additional file 2 — Kimura-corrected average pairwise distances (intersection between line and column) between aligned sequence of Rex3 partial reverse transcriptase sequences from Cichlids and Perciformes marine species. Above diagonal, average genetic distance within species. [file 1471-2148-13-152-S2.pdf]

Additional file 2. Kimura-corrected average pairwise distances (intersection between line and column) between aligned sequence of *Rex*3 partial reverse transcriptase sequences from Cichlids and Perciformes marine species. Above diagonal, average genetic distance within species.

	Cichid species							Perciformes marine species			
	Neotropical						African				
	C. monoculus	*A. ocellatus*	*G. proximus*	*P. scalare*	*S. discus*	*C. labridens*	*O. niloticus*	*Gymnodraco* sp.	*Notothenia* sp.	*Siniperca* sp.	*Cissostichus* sp.
	6.60%	6.80%	9.00%	13.00%	4.40%	4.80%	1.80%	0	0	0	0
C. monoculus											
A. ocellatus	9.11%										
G. proximus	11.31%	10.57%									
P. scalare	15.33%	13.39%	16.35%								
S. discus	11.56%	10.60%	12.58%	13.56%							
C. labridens	10.83%	10.02%	12.49%	13.37%	6.96%						
O. niloticus	13.13%	12.54%	13.94%	18.16%	13.57%	12.59%					
Gymnodraco sp.	31.25%	32.10%	31.60%	37.08%	31.86%	32.67%	29.39%				
Notothenia sp.	29.32%	29.76%	29.68%	34.75%	29.84%	30.61%	27.43%	2.63%			
Siniperca sp.	22.10%	23.29%	22.69%	27.66%	21.52%	22.32%	16.31%	29.56%	27.59%		
Cissostichus sp.	30.02%	30.74%	30.15%	35.64%	30.50%	31.32%	28.10%	2.19%	2.19%	28.26%	
