# Supplementary material for: Evolutionary dynamics of retrotransposable elements Rex1, Rex3 and Rex6 in neotropical cichlid genomes
Source: BMC Evol Biol. 2013 Jul 16;13:152. doi: 10.1186/1471-2148-13-152 (PMC3728117; doi:10.1186/1471-2148-13-152)
Supplement: Additional file 3 — Kimura-corrected average pairwise distances (intersection between line and column) between aligned sequence of Rex6 partial endonuclease from Neotropical and African species. Above diagonal, average genetic distance within species. [file 1471-2148-13-152-S3.pdf]

Additional file 3. Kimura-corrected average pairwise distances (intersection between line and column) between aligned sequence of Rex6 partial endonuclease from Neotropical and African species. Above diagonal, average genetic distance within species.

	Neotropical species							African species	
	C. monoculus	*A. ocellatus*	*G. proximus*	*P. scalare*	*S. discus*	*Crenicichla sp.*	*C. labridens*	*Melanochromis* sp.	*Oreochromis* sp.
	10.60%	5.30%	4.00%	2.30%	10.20%	0	0	0	11.20%
C. monoculus									
A. ocellatus	15.88%								
G. proximus	19.47%	23.74%							
P. scalare	16.33%	19.47%	17.68%						
S. discus	19.10%	24.63%	24.03%	18.37%					
Crenicichla sp.	12.50%	17.33%	12.60%	10.57%	16.46%				
C. labridens	14.41%	19.67%	13.91%	9.89%	16.44%	6.89%			
Melanochromis sp.	16.94%	21.06%	21.67%	17.77%	19.51%	13.58%	14.73%		
Oreochromis sp.	19.63%	23.17%	24.40%	19.46%	22.01%	15.98%	17.62%	6.52%	
